# Supplementary material for: Reproductive Morphology and Success in Annual versus Perennial Legumes: Evidence from Astragalus and the Fabeae (Papilionoideae)
Source: Plants (Basel). 2024 Aug 26;13(17):2380. doi: 10.3390/plants13172380 (PMC11397103; doi:10.3390/plants13172380)
Supplement: Supplementary file 1 [file plants-13-02380-s001.zip › Table S2.pdf]

Table S2. Primary data on productivity and its components in *Astragalus*.

Species	Life cycle	Fruits	Flowers	F/F ratio	Seeds	Ovules	S/O rate	Source (see References in the article)
Astragalus cicer L.	2	12.60	27.70	0.45	4.46	10.92	0.41	[36]
		–	–	0.71	–	–	0.69	[43]
		14.20	18.30	0.78	4.40	–	–	[44]
		17.51	23.70	0.80	–	–	–	[45]
		5.00	26.00	0.19	5.00		–	[46]
		16.31	–	–	5.95	–	–	[47]
A. danicus Retz.	2	7.00	13.00	0.54	2.00	–	–	[46]
		**8.20**	**11.60**	**0.71**	**3.45**	**15.90**	**0.22**	[36]
		–	–	0.63	8.91	18.11	0.49	[32]
		**23.20**	**29.80**	**0.78**	**2.1**	**10.2**	**0.21**	[48]
A. arenarius L.	2	**2.70**	**6.80**	**0.40**	**3.70**	**16.13**	**0.23**	[36]
		**0.71**	**7.00**	**0.10**	**7.00**	**–**	**–**	[47]
A. glycyphyllos L.	2	**1.77**	**21.33**	**0.08**	**11.33**	**–**	**–**	[47]
		11.30	15.20	0.74	16.48	17.93	0.92	[36]
		–	–	0.75	7.00	18.00	0.39	[49]
		9.10	12.60	0.72	8.40	10.20	0.82	[48]
		12.00	–	–	13.88	–	–	[47]
		–	–	–	3.00	14.00	0.21	[50]
A. cornutus Pall.	2	**–**	**–**	**0.40**	**14.10**	**21.40**	**0.66**	[51]
A. falcatus Lam.	2	**40.00**	**70.00**	**0.57**	**8.50**	**12.90**	**0.66**	[51]
		–	–	0.65	7.00	12.00	0.58	[49]
		29.36	–	–	8.99	–	–	[47]
		23.20	30.50	0.76	4.10	–	–	[44]
A. sulcatus L.	2	12.00	20.00	0.60	5.30	10.40	0.51	[51]
		–	7.40	0.04	2.00	–	–	[52]
A. gorczakovskii L.I.Vassiljeva	2	**–**	**–**	**0.54**	**3.98**	**10.60**	**0.38**	[51]
A. onobrychis L.	2	20.00	–	–	6.50	–	–	[47]
		–	–	0.68	6.42	12.80	0.50	[32]
		12.80	20.80	0.62	1.70	–	–	[44]

		15.65	24.20	0.65	–	–	–	[53]
A. ponticus Pall.	2	**50.06**	**53.06**	**0.94**	**1.83**	**9.92**	**0.19**	[54]
		**52.30**	**54.50**	**0.96**	**1.40**	**13.50**	**0.10**	[55]
		48.81	55.98	0.87	1.93	10.67	0.20	[56]
		**66.77**	**79.47**	**0.84**	**1.33**	**13.10**	**0.10**	[57]
		51.90	60.40	0.86	1.50	–	–	[44]
A. olchonensis Gontsch.	2	**5.02**	**–**	**–**	**2.00**	**3.56**	**0.56**	[58]
		**13.40**	**22.80**	**0.59**	**2.60**	**6.20**	**0.42**	[59]
A. exscapus L.	2	**–**	**–**	**0.27**	**3.60**	**12.00**	**0.30**	[60]
A. nitidiflorus Jiménez Mun. & Pau	2	**3.98**	**21.00**	**0.19**	**11.46**	**16.60**	**0.69**	[61]
A. alpinus L.	2	–	–	0.62	–	6.95	0.20	[62]
A. ampullarioides (S.L.Welsh) S.L.Welsh	2	**–**	**–**	**0.10**	**5.90**	**20.40**	**0.32**	[28]
		**–**	**–**	**–**	**–**	**–**	**0.40**	[63cited from 9]
A. holmgreniorum Barneby	2	**–**	**–**	**0.14**	**18.05**	**–**	**0.49**	[28]
		**–**	**–**	**0.38**	**–**	**–**	**–**	[63cited from 9]
A. bozakmanii Podlech	2	**–**	**–**	**–**	**4.58**	**10.00**	**0.46**	[64]
A. argaeus Boiss. & Balansa	2	**–**	**–**	**0.87**	**1.90**	**3.26**	**0.58**	[65]
A. clerceanus Iljin & Krasch.	2	–	–	–	9.40	15.70	0.60	[66]
A. karelinianus Popov	2	–	–	–	12.20	20.60	0.59	[66]
A. sericeocanus Gontsch.	2	**5.99**	**11.23**	**0.65**	**3.72**	**8.23**	**0.45**	[67]
A. kungurensis Boriss. (=*A. wolgensis* Bunge)	2	**1.94**	**2.52**	**0.77**	**10.65**	**21.50**	**0.50**	[68]
A. filicaulis Fisch. & C.A.Mey.	1	–	–	0.69	–	7.10	0.34	[69]
		3.19	7.20	0.44	6.30	–	–	[53]
A. campylorhynchus Fisch. & C.A.Mey.	1	–	–	0.65	–	15.00	0.41	[69]
		–	–	–	15.13 ± 1.46	18.50 ± 1.08	0.81 ± 0.08	Original: AA: Goloskokov, 06.06.59
A. vicarius Lipsky	1	–	–	0.43	–	–	0.18	[69]
A. schmalhausenii Bunge	1	–	–	0.62	–	–	0.20	[69]
		6.43	7.50	0.86	9.80	–	–	[52]

A. oxyglottis Steven ex M.Bieb.	1	–	–	0.48	–	–	0.18	[69]
A. campylotrichus Bunge	1	–	–	0.57	–	–	0.31	[69]
A. amphioxys A.Gray	2	–	–	–	9.20	–	0.52	[70]
A. beckwithii Torr. & A.Gray	2	–	–	–	17.10	–	0.68	[70]
A. calycosus Torr. ex S.Watson	2	–	–	–	–	–	0.48	[71]
A. alopecurus Pall.	2	–	–	–	–	–	**0.20**	[72]
		22.00	–	–	1.50	–	–	[47]
A. cibarius E.Sheld.	2	–	–	–	23.00	–	0.78	[70]
		–	–	0.53	–	–	–	[73]
A. cremnophylax Barneby	2	–	–	**0.61**	–	–	**0.60**	[71]
A. dasyanthus Pall.	2	–	–	–	–	–	**0.35**	[74]
		7.30	14.80	0.49	1.90	–	–	[44]
		–	–	–	–	–	0.45	[75]
A. humillimus A.Gray	2	–	–	–	–	–	**0.32**	[71]
A. jaegerianus Munz	2	**1.30**	**7.15**	**0.18**	**7.35**	**16.5**	**0.42**	[76]
A. lentiginosus Douglas	1	–	–	–	–	–	0.50	[77]
		–	–	–	19.20	–	0.91	[70]
A. linifolius Osterh.	2	–	–	**0.43**	–	–	**0.18**	[33]
A. lonchocarpus Torr.	2	–	–	0.32	–	–	0.26	[33]
A. miser Douglas	2	–	–	–	6.30	–	0.42	[70]
		–	–	0.15	–	–	–	[78cited from 9]
A. nuttallianus DC.	1	–	–	–	14.50	–	0.97	[71]
A. osterhoutii M.E.Jones	2	–	–	**0.20**	–	–	**0.32**	[33]
A. pectinatus (Hook.) G.Don	2	–	–	0.20	–	–	0.45	[33]
A. tennesseensis Chapman	2	–	–	–	**26.70**	**29.67**	**0.90**	[79]
A. troglodytus S.Watson	2	–	–	–	–	–	**0.52**	[71]
A. utahensis (Torr.) Torr. & A.Gray	2	–	–	–	12.80	–	0.48	[70]
		–	–	0.83	–	–	–	[73]
A. ionae Palib.	2	–	–	–	–	–	**0.80**	[80]

A. globiceps Bunge	2	–	–	–	–	–	0.11	[81]
A. galegiformis L.	2	27.00	31.40	0.86	2.00	–	–	[44]
		13.00	–	–	4.00	–	–	[47]
A. canadensis L.	2	20.70	22.10	0.94	3.10	–	–	[44]
		–	–	0.90	–	–	–	[82cited from 9]
A. agnicidus Barneby	2	**–**	**–**	**0.70**	**–**	**–**	**–**	[83]
A. australis (L.) Lam.	2	**3.17**	**15.59**	**0.20**	**2.33**	**13.59**	**0.17**	[84]
A. cymbicarpos Brot.	1	–	–	0.66	–	23.22	–	[25]
A. edulis Bunge	1	–	–	0.24	–	5.93	–	[25]
A. epiglottis L.	1	–	–	0.74	–	4.17	–	[25]
A. filipes A.Gray	2	–	–	0.55	2.71	–	–	[85]
A. hamosus L.	1	–	–	0.31	–	25.1	–	[25]
A. kentrophyta A.Gray	2	–	–	0.31	–	–	–	[78cited from 9]
A. mollissimus Torr.	2	–	–	0.10	–	–	–	[86cited from 9]
A. montii S.L.Welsh	2	**–**	**–**	**0.27**	**–**	**–**	**–**	[78cited from 9]
A. peckii Piper	2	**–**	**–**	**0.28**	**1.57**	**–**	**–**	[87]
A. scaphoides (M.E.Jones) Rydb.	2	**7.40**	**1.26**	**0.17**	**–**	**–**	**–**	[88]
A. tragacantha L.	2	**–**	**–**	**0.43**	**1.75**	**–**	**–**	[89cited from 9]
A. gines-lopezii Talavera, Podlech, Devesa & F.M.Vázquez	2	**1.51**	**3.85**	**0.45**	**11.85**	**–**	**–**	[90]
A. turkestanus Bunge	2	–	3.50	0.56	4.00	–	–	[52]
A. nuciferus Bunge	2	–	3.05	0.68	6.30	–	–	[52]
A. mucidus Bunge	2	–	4.60	0.48	3.40	–	–	[52]
A. floccosifolius Sumnev.	2	–	6.20	0.46	2.50	–	–	[52]
A. schanginianus Pall.	2	–	4.50	0.57	5.40	–	–	[52]
A. substipitatus Gontsch.	2	–	4.60	0.73	7.80	–	–	[52]
A. alopecias Pall.	2	–	55.01	0.46	2.20	–	–	[52]
A. petkoffii B.Fedtsch.	2	–	15.20	0.39	3.60	–	–	[52]
A. platyphyllus Kar. & Kir.	2	–	27.20	0.84	3.20	–	–	[52]

A. fedtschenkoanus Lipsky	2	–	13.05	0.71	8.80	–	–	[52]
A. sulcatus L.	2	–	7.40	0.04	2.00	–	–	[52]
A. lehmannianus Bunge	2	**–**	**–**	**0.76**	**1.95**	**–**	**–**	[91]
A. norvegicus Grauer	2	–	–	0.32	–	–	–	[92]
A. aboriginum var. *richardsonii* (E.Sheld.) B.Boivin	2	–	–	0.37	–	–	–	[92]
A. testiculatus Pall.	2	**–**	**–**	**0.74**	**5.10**	**8.90**	**0.57**	[93]; Prokopyev A.S., personal communication
A. sieversianus Pall.	2	–	–	–	4.00 ± 2.21	15.80 ± 1.32	0.25 ± 0.14	Original: Kazakhstan, ca. 150 km from Taraz to Almaty
A. albicans Bong.	2	**1.40**	**6.78**	**0.19**	**6.76**	**12.20**	**0.56**	[94]
A. vaginatus Pall.	2	**3.44**	**20.75**	**0.17**	**6.20**	**12.60**	**0.50**	[94]
A. pseudoaustralis Fisch. & C.A.Mey.	2	**2.10**	**6.14**	**0.23**	**2.53**	**6.25**	**0.39**	[94]
A. candidissimus Ledeb.	2	**3.60**	**10.53**	**0.33**	**3.65**	**12.65**	**0.25**	[94]
A. veresczaginii Krylov & Sumnev.	2	**10.00**	**11.28**	**0.79**	**4.43**	**15.07**	**0.25**	[95]
A. permiensis C.A.Mey.	2	**2.40**	**2.90**	**0.83**	**7.80**	**18.30**	**0.43**	[96]

Note: 1 = annuals, 2 = perennials, dash = data absent, bold = data obtained from taxa characterized as rare or endangered/threatened. In the case of originally obtained data, these are represented as average ± standard deviation
