# Supplementary material for: Reproductive Morphology and Success in Annual versus Perennial Legumes: Evidence from Astragalus and the Fabeae (Papilionoideae)
Source: Plants (Basel). 2024 Aug 26;13(17):2380. doi: 10.3390/plants13172380 (PMC11397103; doi:10.3390/plants13172380)
Supplement: Supplementary file 1 [file plants-13-02380-s001.zip › Table S3.pdf]

Table S3. Primary data on productivity and its components in the Fabeae

Species	Life cycle	Fruits	Flowers	F/F ratio	Seeds	Ovules	S/O rate	Source (see References in the article)
Lathyrus vernus (L.) Bernh.	2	–	–	–	6.10	14.10	0.43	Original: 55.698209 N, 36.730426 E, 2012
		0.50	7.20	0.07	5.90	11.20	0.53	Original: same locality, 2019
		2.01	8.40	0.24	5.09	13.40	0.38	[29]
		–	–	–	6.33	13.62	0.46	[97]
		2.21 ± 1.57	6.89 ± 1.81	0.32 ± 0.23	–	–	–	Original: 54.726364 N, 37.159582 E, 2022
L. pratensis L.	2	3.60	9.80	0.36	2.00	11.50	0.17	Original: 55.701955 N, 36.727716 E, 2016
		–	–	–	5.50	9.30	0.59	Original: 55.688446 N, 37.278817 E, 2020
		–	–	0.31	–	–	–	[98]
		–	–	–	4.97	10.36	0.48	[97]
L. sylvestris L.	2	–	–	–	5.48	12.09	0.46	[97]
		–	–	–	2.65	12.90	0.21	[34]
		2.60	7.90	0.33	5.20	13.00	0.40	Original: 55.670208 N, 36.719394 E, 2019
		2.40 ± 1.67	11.30 ± 1.38	0.21 ± 0.15	4.90 ± 2.04	9.42 ± 0.70	0.55 ± 0.21	Original: 54.742255 N, 37.187023 E, 2020
L. japonicus subsp. *maritimus* (L.) P.W.Ball	2	4.00	7.10	0.57	6.10	9.60	0.63	Original: 55.327436 N, 20.995064 E, 2019
		–	–	–	6.41	9.31	0.69	[99]
L. latifolius L.	2	–	–	–	6.69	19.44	0.34	[34]
L. gmelinii Fritsch	2	–	–	–	1.50	15.00	0.10	[100]
L. hasslerianus Burkart	2	–	–	–	–	–	0.85	[101]
L. miniatus M. Bieb. ex Steven	2	–	–	–	5.65	9.52	0.60	J.A. Akopian, personal communication, observations in the living collection of Yerevan Botanical Garden, Armenia, 2022
L. niger (L.) Bernh.	2	1.91 ± 0.83	9.36 ± 0.81	0.20 ± 0.08	3.15	9.65	0.32	Original: Botanical garden of Lomonosov Moscow state university, Russia, 2022
L. tuberosus L.	2	2.58 ± 0.99	5.77 ± 0.73	0.44 ± 0.15	1.88 ± 2.40	11.64 ± 1.47	0.15 ± 0.08	Original: 55.6797408 N, 37.803646 E, 2022

		2.10 ± 1.48	5.74 ± 0.87	0.36 ± 0.21	2.05 ± 1.17	13.67 ± 0.71	0.15	Original: 43.234921 N, 76.909344 E, 2022
L. odoratus L.	1	–	–	–	3.40	12.10	0.28	[102]
L. ochrus (L.) DC.	1	1.00 ± 0.00	1.00 ± 0.00	1.00 ± 0.00	2.63 ± 0.92	3.83 ± 0.41	0.69 ± 0.21	Original: Origin unknown, reproduced on experimental plot at Zvenigorod biological station, Russia, 2022
L. sativus L.	1	–	1.00	–	2.43	3.30	0.74	[99]
		–	1.00	0.55	–	–	–	[103]
		–	1.00	–	1.74	–	–	[104]
L. inconspicuus L.	1	–	–	–	5.10	5.70	0.89	[105]
L. hirsutus L.	1	–	–	–	5.00	6.10	0.82	[106]
		–	–	0.90	5.5	6.50	–	[107]
L. cassius Boiss.	1	–	–	0.91	5.65	7.00	0.81	[106]
L. chloranthus Boiss.	1	–	–	0.58	–	9.00	–	[107]
L. aphaca L.	1	–	–	0.80	–	6.00	–	[107]
L. nissolia L.	1	1.00	1.00	1.00	5.00	8.80	0.57	[107, 108]
L. cicera L.	1	–	–	–	3.10	5.40	0.57	[107, 108]
		1.00 ± 0.00	1.00 ± 0.00	1.00 ± 0.00	3.36 ± 0.74	5.00 ± 0.41	0.67 ± 0.14	Original: Armenia (voucher: MW1010150), reproduced on experimental plot at Zvenigorod biological station, Russia, 2022
L. tingitanus L.	1	–	–	1.00	5.75	–	–	[109]
P. sativum L.	1	–	–	–	6.20	7.10	0.87	Original: line ‘SGE’ grown on experimental plot at Zvenigorod biological station, Russia, 2022
P. sativum subsp. *arvense* (L.) Asch. & Graebn.	1	1.05 ± 0.22	1.15 ± 0.37	0.98 ± 0.11	4.14 ± 1.46	5.41 ± 0.80	0.76 ± 0.25	Original: Armenia, reproduced at Zvenigorod biological station, 2022
Vicia sylvatica L.	2	3.94 ± 2.31	14.65 ± 0.92	0.28 ± 0.18	1.73 ± 0.24	4.41 ± 0.01	0.38 ± 0.02	Original: 55.695805 N, 36.723130 E, pooled data from 2017 and 2022
V. benghalensis L.	2	–	–	–	2.56	4.06	0.63	[110]
		–	–	0.22	–	–	–	[11]
V. cracca L.	2	5.15	34.89	0.15	2.1	6.40	0.33	[35]

		–	–	–	2.25	4.94	0.46	[110]
		–	–	–	–	–	0.84	[111]
		–	–	–	–	–	0.86	[112]
		–	–	–	2.28	5.24	0.44	[97]
		–	–	–	2.06	3.76	0.55	[113]
		–	–	0.10	–	–	–	[114]
V. sepium L.	2	–	–	–	5.10	12.70	0.40	[97]
		1.10	4.00	0.28	2.40	13.90	0.17	Original: 55.701067 N, 36.731024 E, 2019
		–	–	–	3.41 ± 1.92	11.79 ± 1.37	0.29 ± 0.17	Original: 55.688446 N, 37.278817 E, 2020
		2.09 ± 1.22	4.30 ± 0.48	0.49 ± 0.28	3.33 ± 1.06	11.78 ± 1.04	0.27 ± 0.12	Original: 54.739528 N, 37.183849 E, 2022
V. alpestris Steven	2	–	–	–	3.00	5.00	0.60	[49]
V. tenuifolia Roth	2	–	–	–	–	–	0.91	[111]
		8,55	22,65	0,37	–	–	–	[115]
V. multicaulis Ledeb.	2	–	–	0.63	–	–	0.33	[116]
V. tsydenii Malyshev	2	0.84	6.86	0.12	–	–	0.43	[117]
		0.53	7.03	0.08	1.60	–	–	[118]
V. pisiformis L.	2	1.00 ± 1.41	43.57 ± 10.97	0.02	3.33 ± 0.58	7.44 ± 0.88	0.45	Original: 54.742322 N, 37.185399 E, 2022
V. unijuga A.Br.	2	2.00 ± 1.63	9.93 ± 3.59	0.20	1.40 ± 0.63	5.60 ± 0.63	0.25 ± 0.12	Original: Botanical garden of Lomonosov Moscow state university, Russia, 2022
V. pubescens (DC.) Link	1	–	–	–	2.87	4.40	0.65	[110]
V. parviflora Cav.	1	–	–	–	3.5	3.92	0.89	[110]
V. monantha Retz.	1	–	–	0.24	–	–	–	[11]
V. articulate Hornem.	1	–	–	0.26	–	–	–	[11]
V. disperma DC.	1	–	–	–	1.97	2.17	0.91	[110]
		–	–	0.10	–	–	–	[11]
V. michauxii Spreng.	1	–	–	0.91	–	–	–	[11]
V. tetrasperma (L.) Schreb.	1	–	–	–	3.11 ± 0.90	4.18 ± 0.59	0.76 ± 0.22	Original: 54.748894 N, 37.190894 E, 2022

		1.10	1.10	1.00	3.60	4.20	0.86	Original: 55.703080 N, 36.731651 E, 2019
V. lutea L.	1	–	–	–	2.81	5.17	0.54	[110]
		–	–	0.63	–	–	–	[11]
V. lutea subsp. *vestita* (Boiss.) Rouy	1	–	–	–	2.57	3.77	0.68	[110]
V. hirsuta (L.) Gray	1	–	–	–	1.75 ± 0.44	2.00 ± 0.00	0.87 ± 0.22	Original: 54.748894 N, 37.190894 E, 2022
		3.5	3.75	0.93	1.90	2.00	0.95	Original: 55.702092 N, 36.728335 E, 2019
		–	–	–	1.50	1.86	0.81	[110]
		–	–	0.60	–	–	–	[11]
V. bithynica (L.) L.	1	–	–	0.41	–	–	–	[11]
V. anatolica Turrill	1	–	–	0.42	–	–	–	[11]
V. faba L.	1	–	3.70	–	2.50	2.90	0.86	Original: cv. 'Russkiye Chyornye', grown on experimental plot at Zvenigorod biological station, Russia, 2020
		–	–	0.22	–	–	–	[103]
		–	–	–	3.18	4.00	0.79	[119]
		–	–	0.07	–	–	–	[103]
V. narbonensis L.	1	–	–	0.63	–	–	–	[11]
V. vicioides (Desf.) Cout.	1	–	–	–	1.70	2.03	0.84	[110]
V. sativa L.	1	1.10	1.20	0.94	6.40	10.00	0.64	Original: 55.507893 N, 31.088042 E, 2016
		0.71 ± 0.46	1.00 ± 0.00	0.71 ± 0.46	6.92 ± 2.02	8.75 ± 1.06	0.82 ± 0.19	Original: 54.743755 N, 37.190057 E, 2022
		–	–	–	5.25	6.80	0.75	[110]
V. sativa subsp. *nigra* (L.) Ehrh.	1	–	–	–	10.22	11.54	0.89	[110]
		–	–	0.32	–	–	–	[11]
V. sativa subsp. *cordata* (Hoppe) Asch. & Graebn.	1	–	–	0.72	–	–	–	[11]
V. sativa subsp. *macrocarpa* (Moris) Arcang.	1	–	–	0.54	–	–	–	[11]
V. hybrida L.	1	–	–	0.19	–	–	–	[11]

V. villosa Roth		–	–	0.11	5.30	8.15	0.65	[120]
	1	3.09 ± 1.20	10.60 ± 2.23	0.29	2.30 ± 1.00	5.63 ± 0.12	0.45 ± 0.20	Original: 54.924671 N, 36.889372 E, 2022
		–	–	–	2.79	–	–	[121]
V. villosa subsp. *varia* (Host) Corb.	1	–	–	–	2.55	4.10	0.62	[110]
		–	–	–	3.31	4.31	0.77	
V. peregrina L.		–	–	–	2.93	5.87	0.5	[110]
	1	1.00 ± 0.00	1.00 ± 0.00	1.00 ± 0.00	3.93 ± 1.14	5.17 ± 0.70	0.78 ± 0.18	Original: Armenia (voucher: ERE151319), reproduced on experimental plot at Zvenigorod biological station, Russia, 2022
				0.73				[11]
V. ervilia (L.) Willd.	1	1.13 ± 0.35	1.13 ± 0.35	1.00 ± 0.00	2.26 ± 0.87	3.15 ± 0.59	0.71 ± 0.26	Original: Origin unknown, reproduced on experimental plot at Zvenigorod biological station, Russia, 2022
V. hyrcanica Fisch. & C.A.Mey.	1	–	–	0.08	–	–	–	[11]
V. pannonica Crantz		–	–	–	4.32	–	–	[122]
	1	–	3.80 ± 0.41	–	–	–	–	Original: cv. ‘Prima’, grown at Rimski Šančevi experiment field of Institute of Field and Vegetable Crops, Novi Sad, Serbia, 2023
V. pannonica var. *purpurascens* Ser.	1	–	–	–	1.89	–	–	[122]
V. grandiflora Scop.	1	1.40 ± 0.50	2.00 ± 0.00	0.70 ± 0.25	10.53 ± 3.38	17.47 ± 1.55	0.59 ± 0.16	Original: 45.237520 N, 19.825829 E, 2023
Lens culinaris Medik.	1	1.90 ± 0.31	1.95 ± 0.22	0.98 ± 0.11	1.73 ± 0.51	2.05 ± 0.23	0.84 ± 0.23	Original: cv. ‘Petrovskaya 4105’, grown on experimental plot at Zvenigorod biological station, Russia, 2022

Note: 1 = annuals, 2 = perennials, dash = data absent. In the case of originally obtained data, these are represented as average ± standard deviation, where available.
